# Psychological Factors Associated with Learning in Bioscience Courses Among Undergraduate Nursing Students

**DOI:** 10.3390/nursrep16070221

**Published:** 2026-06-26

**Authors:** Kyriakos Kiourtidis, Andrea Paola Rojas Gil, Athina Patelarou, Sotirios G. Zarogiannis, Erasmia Rouka

**Affiliations:** 1 Department of Nursing, School of Health Sciences, University of Thessaly, GAIOPOLIS, 41500 Larissa, Greece; kkiourtidis@uth.gr; 2 Laboratory of Basic Health Sciences, Department of Nursing, Faculty of Health Sciences, University of the Peloponnese, Akadimaikou GK, 3 Building OAED, 22100 Tripoli, Greece; arojas@uop.gr; 3 Laboratory of Evidence-Based Healthcare, Education and Clinical Protocols, Department of Nursing, School of Health Sciences, Hellenic Mediterranean University, 71410 Heraklion, Greece; apatelarou@hmu.gr; 4 Department of Physiology, Faculty of Medicine, School of Health Sciences, University of Thessaly, BIOPOLIS, 41500 Larissa, Greece; szarog@uth.gr

**Keywords:** achievement goal theory, bioscience education, perceived stress, quantitative research, self-esteem, undergraduate nursing students

## Abstract

**Background/Objectives**: Undergraduate nursing students consider bioscience courses essential to their education and clinical practice, yet they often find them challenging. This study explored the associations among achievement goal orientations, perceived stress, and self-esteem to examine factors associated with bioscience learning in nursing education. **Methods**: A quantitative cross-sectional study was conducted with undergraduate nursing students enrolled in the first-year courses “Biology–Clinical Biochemistry” and “Genetics”. Data were gathered using the Perceived Stress Scale-14 (PSS14); the Achievement Goal Questionnaire-Revised (AGQ-R), divided into four subscales, each representing a goal type (mastery-approach or AGQ MA, mastery-avoidance or AGQ MAV, performance-approach or AGQ PA, performance-avoidance or AGQ PAV); and the Rosenberg Self-Esteem Scale (RSES). Univariate and multiple regression analyses were conducted using SPSS v26.0, with significance set at *p* < 0.05. **Results**: Perceived stress was negatively associated with self-esteem in both Biology–Clinical Biochemistry and Genetics (*p* < 0.001). The assessment of potential links between quantitative variables and the study topic using univariate analysis showed an association of course category with the PSS14 score (*p* = 0.008). This finding remained significant in the regression analysis (*p* = 0.004), which also identified an effect of gender on the same scale (*p* = 0.029). Multiple regression further revealed associations between AGQ MA and the subject of study (*p* = 0.047), AGQ MAV and gender (*p* = 0.001), AGQ PAV and gender (*p* = 0.016), and RSES Total and type of secondary education (*p* = 0.007). **Conclusions**: Psychological factors interact dynamically with the demands of bioscience education within tertiary nursing curricula, varying according to demographic and academic traits.

## 1. Introduction

Undergraduate nursing students view bioscience courses as vital to their education [1]. These courses are key in shaping nurses’ professional identities and clinical skills. However, many studies [2,3] indicate that students find these courses particularly challenging to understand, especially compared to other subjects. In fact, engaging with biosciences often results in increased stress, discouragement, and reduced self-esteem [4]. Several factors are believed to impact this, including age, year of study, previous knowledge, personal learning goals, mental resilience, teaching methods, opportunities to apply biosciences in clinical practice and research, and the complexity of the material [5]. Therefore, it is crucial to research how biosciences are taught in undergraduate nursing programs. Although students acknowledge the importance of biosciences for developing nursing skills, the difficulties they encounter hinder their ability to learn and integrate knowledge at the academic, research, and professional levels.

The theoretical model, which is gradually developing in learning motivation research, is based on Achievement Goal Theory, which continues to inspire researchers and attract interest in the literature. Assuming that human motivation influences goals that guide behavior [6], this framework initially classifies students’ goals as mastery goals and performance goals. Mastery goals focus on understanding, becoming familiar with, and mastering the subject, while performance goals involve comparing one’s abilities to those of others [7]. However, practical applications of the theory [8] revealed the need for a more detailed differentiation of specific goals, ensuring that the variability in achievement goal orientations observed among students aligns with the model’s theoretical foundation. As a result, goals are divided into mastery-approach goals, which aim to develop new skills and expand knowledge; mastery-avoidance goals, where individuals try to hide incomplete mastery and misunderstanding; performance-approach goals, focused on demonstrating abilities to earn positive evaluations; and performance-avoidance goals, which involve avoiding appearing inadequate compared to others [9,10]. These components form the 2 × 2 model of Achievement Goal Theory, initially supported by Elliot and Murayama in 2008 [11], which has influenced research [12] and led to the development of the revised Achievement Goal Questionnaire (AGQ-R) for practical assessment.

The achievement goal framework has seen limited application among nursing students, highlighting the need for further research into the achievement goals they develop throughout their studies and how these goals relate to Bachelor of Science nursing curricula. Furthermore, the existing literature does not provide a clear or consistent understanding of the relationship between achievement goals and stress among undergraduate nursing students. Nevertheless, available evidence [6,8] suggests that certain goal orientations—particularly mastery-avoidance, performance-approach, and performance-avoidance goals—may, under specific circumstances, be associated with elevated stress levels. These findings underscore the need to further investigate the interplay between achievement goal orientations and stress in nursing education.

Since the outcomes of achievement goals that individuals set greatly influence their self-perception [2], scientific research has shifted toward examining self-esteem in relation to personal goal setting. According to Rosenberg [13,14], self-esteem refers to the positive or negative attitudes a person holds toward themselves, and it is not innate; rather, it develops from the integration of experiences and interactions within their social and psychological environment. The literature [15] emphasizes the vital role of individual goals in the academic context as a means of seeking meaning and personal growth. All these factors collectively shape self-esteem, which is actively expressed through the development of learning or performance goals within the educational setting. Research [16] shows that elevated stress levels are, overall, associated with lower self-esteem.

Moreover, learning-approach goals and performance-approach goals are associated with positive self-esteem perceptions, as individuals focus on personal improvement, whereas learning-avoidance goals and performance-avoidance goals are linked to negative self-esteem outcomes, as motivation centers on avoiding failure to protect the self. Based on current data, this interaction remains largely unexplored among undergraduate nursing students, especially regarding their engagement with bioscience courses.

Given the limited evidence on the interplay among achievement goal orientations, perceived stress, and self-esteem in undergraduate nursing students in bioscience courses, the present study examines these psychological factors simultaneously within bioscience education. This study was guided by several hypotheses. Self-esteem was expected to be negatively associated with perceived stress. Mastery-approach and performance-approach achievement goals were hypothesized to be positively associated with self-esteem and negatively associated with perceived stress. In contrast, mastery-avoidance and performance-avoidance achievement goals were expected to be positively associated with perceived stress and negatively associated with self-esteem. Furthermore, drawing on the broader theoretical framework, it was tentatively hypothesized that students graduating from vocational high schools would report higher perceived stress, lower self-esteem, and greater endorsement of mastery-avoidance and performance-avoidance goals. Similarly, students enrolled in the Genetics course were expected to exhibit higher perceived stress, lower self-esteem, and greater endorsement of mastery-approach and performance-approach goals. No specific hypotheses were formulated regarding the effects of gender, age, and semester of study, given the inconsistent evidence concerning their associations with perceived stress, self-esteem, and achievement goals among undergraduate nursing students.

According to the existing literature [1,2], it is important to examine the challenges students face and highlight issues relevant to tertiary education in nursing. This can help identify problems and inform strategies for future improvements.

## 2. Materials and Methods

### 2.1. Participants and Sampling Procedure

Participants eligible for inclusion were undergraduate nursing students enrolled in the first-year courses “Biology–Clinical Biochemistry” and “Genetics” during the 2023–2024 and 2024–2025 academic years. According to the department’s study guide, “Biology–Clinical Biochemistry” is a mandatory course, while “Genetics” is one of several elective courses available to students to fulfill the program’s elective requirement. The inclusion of these specific courses was justified by their classification within the bioscience field, supported by extensive research [2,17]. Besides basic demographic information (gender, age, students’ progression within the academic program, semester, type of secondary education, general or vocational high school), students’ participation did not require access to any other personal data. Data collection took place during the penultimate lecture of each course. The timing of data collection was intended to ensure sufficient exposure to course content before the assessment of achievement goals, perceived stress, and self-esteem. Additionally, previous research [18] indicates that students experience significantly higher stress levels during the final exam period than at other times in the semester.

At the start of the second-to-last lecture of the semester, the researcher and supervising professor clearly presented the purpose of this research to the students and explained that participation is completely voluntary. Those who agreed signed informed consent forms that outlined the entire research process. Participants were assured that confidentiality and privacy policies required all data to be fully anonymized. The signed consent forms were stored in the lead researcher’s records. In addition to the information sheet and consent process, all participants were given the opportunity to learn about the research results. This research received approval from the Ethics Committee of the Department of Nursing, School of Health Sciences, University of Thessaly, Greece (08-06-2023/698).

### 2.2. Data Collection Method

To evaluate the perceived stress levels among students, the “Perceived Stress Scale-14” or “PSS14” was used. This scale was created by Cohen, Kamarck, and Mermelstein in 1983 [19]. The items on the scale measure how much individuals see their lives as unpredictable, uncontrollable, or overwhelming, with responses recorded on a Likert scale from 0 (never) to 4 (very often). The scale has been translated and validated for the local population [20,21]. After coordinating with the research team that adapted the scale, permission for use and scoring instructions were obtained. According to the literature, the PSS-14 is a reliable and valid tool, as demonstrated in studies involving diverse populations [22].

The achievement goals in the academic setting were measured using the “Achievement Goal Questionnaire-Revised” or “AGQ-R,” developed by Elliot and Murayama in 2008 [11]. The questionnaire is divided into four subscales, each representing a goal type (mastery-approach or “AGQ MA,” mastery-avoidance or “AGQ MAV,” performance-approach or “AGQ PA,” performance-avoidance or “AGQ PAV”), with three items per subscale. Responses are recorded on a Likert scale ranging from 1 (strongly disagree) to 5 (strongly agree). The questionnaire has been translated and standardized for the population [23]. Additionally, communication was established with the research team responsible for adapting the tool to the local population, and permission to use it, along with the relevant instructions, was granted. According to the literature, the AGQ-R demonstrates good sensitivity and validity, although the overall score varies significantly across different populations [24,25].

To assess participants’ self-reported self-esteem, the “Rosenberg Self-Esteem Scale” or “RSES” was used. Developed by Rosenberg in 1965 [13], this unidimensional tool assesses an individual’s self-esteem from their perspective, with responses recorded on a 4-point Likert scale from 1 (strongly disagree) to 4 (strongly agree). The scale has been translated and adapted for the local population [26,27]. Furthermore, contact was made with the authors who adapted the instrument locally, and permission for use, along with the evaluation instructions, was granted. The tool has demonstrated good reliability and validity, as confirmed by multiple studies with diverse populations [28].

### 2.3. Statistical Analysis

Statistical analysis was conducted using SPSS v26.0 (IBM, Armonk, NY, USA). The primary analyses examined associations among perceived stress, self-esteem, and achievement goal orientations. Reliability indices, as reflected by Cronbach’s alpha, were calculated with scale analysis. The Kolmogorov–Smirnov test was employed to assess the distribution of the data. Relationships between quantitative variables were assessed with either the Pearson (r) or Spearman (ρ) correlation coefficient, depending on the data. The independent-samples *t*-test and the Mann–Whitney test were used to identify significant differences between two groups in parametric and nonparametric data, respectively. Multiple linear regression was used to assess the impact of all explanatory variables on the scales studied, with all variables entered simultaneously (method: Enter). Given the exploratory nature of several analyses, no formal adjustment for multiple comparisons was applied. For descriptive statistics, continuous variables are presented as the mean ± standard error of the mean (SEM), median, and standard deviation (SD). Statistical significance was established at *p* < 0.05.

## 3. Results

The number of eligible students was 370, of whom 155 declined to participate. A total of 215 undergraduate nursing students participated in this study and completed the questionnaires without missing data (41 males, 173 females, and 1 non-binary; response rate 58.1%). Gender was coded as male/female; because there was a single observation in the non-binary category, this case was excluded from analyses in which gender was included as an explanatory variable but retained in descriptive reporting, to ensure that their responses were represented in the study findings. Cronbach’s alpha was equal to 0.851 for PSS14, 0.781 for AGQ-R, and 0.878 for RSES. Descriptive statistics for all subscales and scales by study subject are presented in Table 1.

### 3.1. Biology–Clinical Biochemistry Module

Univariate analysis revealed significant associations between AGQ MAV and gender, RSES and type of secondary education, and AGQ PA and students’ progression within the academic program in terms of semester (Mann–Whitney *p* = 0.014; *p* = 0.007; *p* = 0.022, respectively). These associations remained significant in the multiple regression analysis (*p* = 0.025; *p* = 0.018; *p* = 0.044). Correlations among all quantitative variables are shown in Table 2.

### 3.2. Genetics Module

Univariate analysis revealed significant associations between AGQ MAV, AGQ PAV, and gender, as well as between AGQ PA, AGQ PAV, and type of secondary education (Mann–Whitney *p* = 0.004, *p* = 0.029, *p* = 0.004, and *p* = 0.011, respectively). An association between PSS14 and gender was also identified (*t*-test, *p* = 0.009). Multiple regression analysis confirmed these associations (*p* = 0.005; *p* < 0.001; *p* = 0.002; *p* = 0.004; *p* = 0.013). It also revealed significant associations of RSES with age (*p* = 0.023) and type of secondary education (*p* = 0.023). Correlations among all quantitative variables are shown in Table 3.

### 3.3. Biology–Clinical Biochemistry/Genetics Modules Combined

**Figure 1:**
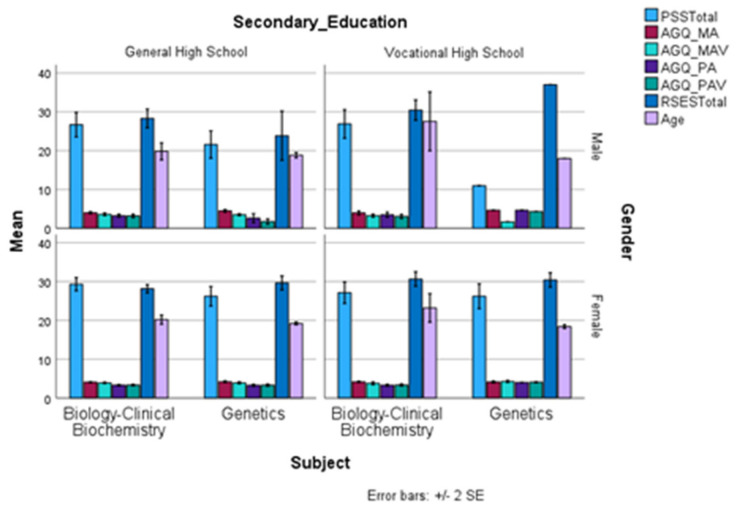
Mean scores of perceived stress (PSS-14), achievement goals (AGQ-R subscales), self-esteem (RSES), and age according to course (Biology–Clinical Biochemistry and Genetics), gender, and type of secondary education. Error bars represent ±2 standard errors of mean (SEMs).

The assessment of potential links between quantitative variables and the study topic using univariate analysis showed an association of course category with the PSS14 score (*t*-test *p* = 0.008). This finding remained significant in the regression analysis (*p* = 0.004), which also identified an effect of gender on the same scale (*p* = 0.029). Multiple regression further revealed associations between AGQ MA and the subject of study (*p* = 0.047), AGQ MAV and gender (*p* = 0.001), AGQ PAV and gender (*p* = 0.016), and RSES Total and type of secondary education (*p* = 0.007) (Figure 1). The results of multiple regression analyses are presented in Table 4.

## 4. Discussion

The academic paths of nursing students are shaped by various factors. The main aim of this study was to examine the associations among self-esteem, perceived stress, and achievement goal orientations among undergraduate nursing students enrolled in bioscience courses. The data analysis revealed a clear pattern in how these variables are related and interact. Additionally, individual characteristics, such as age, sex, and prior academic experience, were related to the measures examined.

Female sex was consistently associated with higher mastery-avoidance goals in both courses and with performance-avoidance goals in Genetics. This clearly shows that female students are more likely to avoid revealing a lack of understanding and worry about appearing inadequate in the learning environment. Simultaneously, the higher PSS-14 scores within the same group indicate increased stress. This pattern contrasts with findings from other studies that report no gender differences in stress levels [29,30]. Nonetheless, the presence of mastery-avoidance and performance-avoidance goals among women is indirectly linked to higher stress, as fears and concerns about inadequacy in educational settings are primary drivers of these goals. Similarly, the literature [31,32] confirms that mastery-avoidance and performance-avoidance goals are associated with higher perceived stress among students.

Conversely, students from vocational high schools demonstrated significantly higher performance-approach and performance-avoidance goals in Genetics, indicating that they may focus heavily on demonstrating their abilities to receive positive feedback and on avoiding the appearance of inadequacy in the learning environment. At the same time, this group also scored higher on the RSES in both courses, reflecting a higher level of self-perception. The presence of increased self-esteem in a group that expresses stronger performance-approach and performance-avoidance goals aligns with the nature of these goals, which are mainly based on external incentives and the validation of personal effort. Relevant research [31,33] supports this tendency and emphasizes the increased stress that often accompanies performance-approach and performance-avoidance goals.

Additionally, students’ age was associated with the RSES tool in Genetics, specifically their self-esteem. The existing literature agrees with this finding, noting that age affects various aspects of student life related to stress development and the smooth progress of academic studies [30], which may indicate that age intertwines with adaptation to academic demands, as well as previous educational experiences. Students’ progression within the academic program (semester) was also found to be significant among first-year nursing students in Biology–Clinical Biochemistry, who showed greater performance-approach goals, reflecting a stronger motivation to demonstrate their abilities and gain positive feedback within the educational environment. The challenges faced by undergraduate nursing students transitioning from secondary education to university are well documented, as they tend to experience increased stress and higher goal striving at the start of their academic journey [32]. Among the two courses, Genetics was associated with higher scores on mastery-approach goals. This suggests that students enrolled in Genetics may experience a stronger desire to learn new knowledge than those in Biology–Clinical Biochemistry. Since Genetics is an elective course, it is understandable that it attracts students driven by internal learning motives for expanding their personal knowledge base. However, given the exploratory nature of subgroup analyses, this finding should be interpreted cautiously and warrants further investigation.

A key correlation from both courses’ data involves the negative relationship between the PSS-14 and the RSES tools. Essentially, this shows that as perceived stress decreases, self-esteem increases and vice versa. This finding aligns with the literature [29,34], which also highlights this contrast between the variables. Additionally, the PSS-14 was positively linked to mastery-avoidant goals in Genetics, indicating that the more students feel the need to hide an insufficient understanding, the more stressed they might become. This interaction underscores the stress related to students’ desire to avoid seeming deficient in the knowledge they are expected to master. Regarding individual learning goals, mastery-approach goals were positively correlated with all other goal types in Biology–Clinical Biochemistry, suggesting that the desire to learn can coexist with various motivations and aspirations. Notably, mastery-avoidance goals showed a positive association with both performance-approach and performance-avoidance goals, meaning that as the avoidance of inadequate comprehension increases, so does the motivation to demonstrate one’s abilities and the desire to avoid appearing incompetent. This complex pattern might be explained by the idea that a shared fear of personal learning inadequacy drives both stress and the need for positive evaluations to lessen this worry. Furthermore, the recent literature [35,36] highlights the complex interactions among these goals, hinting at the dysfunctional aspects they may sustain in learning. This is further supported by the positive correlation observed between performance-approach and performance-avoidance goals across the two courses. This relationship indicates that the desire to showcase one’s abilities can coexist with fears of underperforming relative to others.

From an educational perspective, the findings suggest that bioscience teaching may benefit from strategies designed to reduce perceived stress and promote adaptive learning. Such approaches may include scaffolded learning activities, greater integration of clinical examples into bioscience teaching, structured feedback mechanisms, peer-assisted learning, and academic mentoring. These interventions may help students engage more confidently with bioscience content while reducing concerns related to academic performance and evaluation.

### Study Limitations and Recommendations for Further Research

Although this research addresses the need to examine achievement goals, perceived stress, and self-esteem among undergraduate nursing students in the field of biosciences, it should be interpreted with certain limitations in mind. First, the following points should be considered: a cross-sectional design, potential self-report bias, and gender imbalance. An additional limitation relates to the number of statistical comparisons performed. In addition to the primary hypothesis-driven analyses, several exploratory analyses were conducted. Because no formal correction for multiple testing was applied, the possibility of Type I error cannot be excluded. Therefore, findings with marginal statistical significance should be interpreted cautiously and require confirmation in future studies. Also, the Genetics course included fewer participants than the Biology–Clinical Biochemistry course. Additionally, since this study involves individual psychological factors, personal characteristics are likely to significantly influence their expression within the broader population. Another key limitation is that the sample was drawn from a single Nursing Department, so generalizations should be made cautiously. It would be especially helpful to study these variables in other elective courses with larger enrollments to better understand their specific interactions. Since this study focused on bioscience courses for first-year students, exploring students enrolled in later-semester bioscience courses—where achievement goals might be affected by their academic progression—would also be valuable. Furthermore, the relationship between achievement goals and different teaching methods in bioscience courses remains relatively unclear.

Given that academic performance indicators were not collected in this study protocol, future research should incorporate objective academic outcomes, such as grades, examination scores, and progression indicators, to assess potential associations between psychological variables and academic achievement.

## 5. Conclusions

This study’s findings underscore a range of psychological factors associated with nursing students’ academic progression in bioscience courses. These factors interact dynamically with the demands of bioscience education within tertiary nursing curricula, varying with demographic and academic characteristics. Overall, heightened stress in these courses was confirmed, and female participants in the sample exhibited comparatively higher levels of emotional strain. Furthermore, a dystonic relationship between stress and self-esteem was consistently observed across all measurements conducted in this study. With regard to achievement goals, mastery-approach goals were identified only in the course classified as an elective, whereas mastery-avoidance, performance-approach, and performance-avoidance goals—traditionally associated with elevated stress—were predominant. These findings suggest that future research should investigate whether targeted educational interventions may help reduce stress and support adaptive achievement goals among nursing students enrolled in bioscience courses.

## Figures and Tables

**Table 1:** Descriptive characteristics of participants’ psychological measures and age, by course (N = 215; 165 Biology–Clinical Biochemistry, 50 Genetics).

Subject		PSS14	AGQ_MA	AGQ_MAV	AGQ_PA	AGQ_PAV	RSES *	Age
Biology–Clinical Biochemistry	Mean	28.40	4.0688	3.8000	3.3013	3.3070	28.76	21.08
	Std. Error of Mean	0.631	0.04425	0.06333	0.06716	0.06858	0.414	0.566
	Median	28.00	4.0000	4.0000	3.3300	3.3300	29.00	19.00
	Std. Deviation	8.102	0.56835	0.81352	0.86273	.88093	5.323	7.273
Genetics	Mean	24.94	4.2538	3.7862	3.3002	3.2126	29.20	19.02
	Std. Error of Mean	1.051	0.08286	0.11366	0.14011	0.14803	0.856	0.135
	Median	24.50	4.3300	4.0000	3.6700	3.0000	30.00	19.00
	Std. Deviation	7.432	0.58588	0.80367	0.99076	1.04673	6.054	0.958
Total	Mean	27.60	4.1118	3.7968	3.3011	3.2851	28.86	20.60
	Std. Error of Mean	0.550	0.03931	0.05520	0.06081	0.06277	0.374	0.439
	Median	27.00	4.0000	4.0000	3.3300	3.3300	30.00	19.00
	Std. Deviation	8.069	0.57644	0.80939	0.89172	0.92043	5.490	6.442

* PSS14, Perceived Stress Scale-14; AGQ MA, Mastery-approach goals; AGQ MAV, Mastery-avoidance goals; AGQ PA, Performance-approach goals; AGQ PAV, Performance-avoidance goals; RSES, Rosenberg Self-Esteem Scale.

**Table 2:** Correlation coefficients among psychological variables and age in students enrolled in Biology–Clinical Biochemistry (N = 165).

	Spearman’s Rho	Significance (2-Tailed)	95% Confidence Intervals (2-Tailed)	
			Lower	Upper
PSS14—AGQ_MA *	−0.067	0.395	−0.222	0.092
PSS14—AGQ_MAV	−0.029	0.716	−0.185	0.129
PSS14—AGQ_PA	0.016	0.834	−0.141	0.173
PSS14—AGQ_PAV	0.035	0.656	−0.123	0.191
**PSS14—RSES**	**−0.512**	**<0.001**	**−0.619**	**−0.386**
PSS14—Age	−0.019	0.804	−0.176	0.138
**AGQ_MA—AGQ_MAV**	**0.422**	**<0.001**	**0.283**	**0.543**
**AGQ_MA—AGQ_PA**	**0.413**	**<0.001**	**0.274**	**0.536**
**AGQ_MA—AGQ_PAV**	**0.282**	**<0.001**	**0.130**	**0.420**
AGQ_MA—RSES	0.053	0.496	−0.105	0.209
AGQ_MA—Age	−0.019	0.810	−0.176	0.139
AGQ_MAV—AGQ_PA	0.044	0.574	−0.114	0.200
**AGQ_MAV—AGQ_PAV**	**0.165**	**0.034**	**0.008**	**0.314**
AGQ_MAV—RSES	0.118	0.132	−0.040	0.270
AGQ_MAV—Age	0.066	0.401	−0.092	0.221
**AGQ_PA—AGQ_PAV**	**0.633**	**<0.001**	**0.528**	**0.719**
AGQ_PA—RSES	0.045	0.566	−0.113	0.201
AGQ_PA—Age	−0.063	0.423	−0.218	0.095
AGQ_PAV—RSES	0.049	0.530	−0.109	0.205
AGQ_PAV—Age	−0.038	0.624	−0.195	0.119
RSES—Age	0.032	0.684	−0.126	0.188

* PSS14, Perceived Stress Scale-14; AGQ MA, Mastery-approach goals; AGQ MAV, Mastery-avoidance goals; AGQ PA, Performance-approach goals; AGQ PAV, Performance-avoidance goals; RSES, Rosenberg Self-Esteem Scale. Bold is to highlight statistically significant associations.

**Table 3:** Correlation coefficients among psychological variables and age in students enrolled in Genetics (N = 50).

	Spearman’s Rho	Significance (2-Tailed)	95% Confidence Intervals (2-Tailed)	
			Lower	Upper
PSS14—AGQ_MA *	−0.103	0.477	−0.378	0.189
**PSS14—AGQ_MAV**	**0.424**	**0.002**	**0.157**	**0.633**
PSS14—AGQ_PA	−0.017	0.907	−0.302	0.271
PSS14—AGQ_PAV	−0.138	0.340	−0.408	0.154
**PSS14—RSES**	**−0.471**	**<0.001**	**−0.667**	**−0.213**
PSS14—Age	0.032	0.826	−0.257	0.315
**AGQ_MA—AGQ_MAV**	**0.337**	**0.017**	**0.056**	**0.568**
**AGQ_MA—AGQ_PA**	**0.421**	**0.002**	**0.154**	**0.631**
AGQ_MA—AGQ_PAV	−0.023	0.877	−0.307	0.265
AGQ_MA—RSES	0.205	0.154	−0.086	0.464
AGQ_MA—Age	0.096	0.509	−0.196	0.372
**AGQ_MAV—AGQ_PA**	**0.297**	**0.036**	**0.012**	**0.538**
**AGQ_MAV—AGQ_PAV**	**0.295**	**0.038**	**0.009**	**0.536**
AGQ_MAV—RSES	−0.164	0.256	−0.430	0.128
AGQ_MAV—Age	−0.014	0.923	−0.299	0.273
**AGQ_PA—AGQ_PAV**	**0.558**	**<0.001**	**0.323**	**0.728**
AGQ_PA—RSES	0.142	0.325	−0.150	0.411
AGQ_PA—Age	0.029	0.842	−0.259	0.312
AGQ_PAV—RSES	0.267	0.061	−0.021	0.514
AGQ_PAV—Age	−0.179	0.215	−0.442	0.113
RSES—Age	0.207	0.150	−0.085	0.465

* PSS14, Perceived Stress Scale-14; AGQ MA, Mastery-approach goals; AGQ MAV, Mastery-avoidance goals; AGQ PA, Performance-approach goals; AGQ PAV, Performance-avoidance goals; RSES, Rosenberg Self-Esteem Scale. Bold is to highlight statistically significant associations.

**Table 4:** Multiple regression analyses results (only statistically significant findings are presented).

**Biology–Clinical Biochemistry** (variables were coded as gender: male = 1, and female = 2; semester: 1st semester = 1, and >1st semester = 2; secondary education: general high school = 1, and vocational high school = 2).								
Model		Unstandardized Coefficients		Standardized Coefficients	t	*p*-value	95.0% Confidence Interval for B	
		B	Std. Error	Beta			Lower Bound	Upper Bound
1	(Constant)	3.231	0.441		7.324	<0.001	2.360	4.102
	Gender	0.362	0.160	0.177	2.263	**0.025**	0.046	0.678
a. Dependent Variable: AGQ_MAV; F = 1.854; R square = 0.045; Adjusted R square = 0.021; model *p*-value = 0.121								
1	(Constant)	3.954	0.470		8.404	<0.001	3.025	4.883
	Semester	−0.412	0.203	−0.169	−2.031	**0.044**	−0.812	−0.011
a. Dependent Variable: AGQ_PA; F = 1.324; R square = 0.032; Adjusted R square = 0.008; model *p*-value = 0.263								
1	(Constant)	24.585	2.893		8.499	<0.001	18.872	30.298
	Secondary_Education	2.413	1.013	0.193	2.382	**0.018**	0.412	4.413
a. Dependent Variable: RSESTotal; F = 1.699; R square = 0.041; Adjusted R square = 0.017; model *p*-value = 0.153								
**Genetics** (variables were coded as gender: male = 1, and female = 2; secondary education: general high school = 1, and vocational high school = 2).								
1	(Constant)	23.405	22.515		1.040	0.304	−21.916	68.725
	Gender	6.832	2.646	0.357	2.582	**0.013**	1.505	12.159
a. Dependent Variable: PSSTotal; F = 2.738; R square = 0.152; Adjusted R square = 0.096; model *p*-value = 0.054								
1	(Constant)	3.725	2.406		1.548	0.128	−1.119	8.568
	Gender	0.825	0.283	0.398	2.917	**0.005**	0.256	1.394
a. Dependent Variable: AGQ_MAV; F = 3.165; R square = 0.171; Adjusted R square = 0.117; model *p*-value = 0.033								
1	(Constant)	−3.845	2.884		−1.334	0.189	−9.650	1.959
	Secondary_Education	1.302	0.389	0.461	3.348	**0.002**	0.519	2.085
a. Dependent Variable: AGQ_PA; F = 4.244; R square = 0.217; Adjusted R square = 0.166; model *p*-value = 0.010								
1	(Constant)	1.115	2.820		0.396	0.694	−4.560	6.791
	Gender	1.222	0.331	0.453	3.687	**<0.001**	0.555	1.889
	Secondary_Education	1.165	0.380	0.390	3.063	**0.004**	0.399	1.931
a. Dependent Variable: AGQ_PAV; F = 7.522; R square = 0.329; Adjusted R square = 0.285; model *p*-value < 0.001								
1	(Constant)	−22.170	17.952		−1.235	0.223	−58.305	13.964
	Age	2.108	0.894	0.334	2.358	**0.023**	0.308	3.908
	Secondary_Education	5.710	2.422	0.331	2.358	**0.023**	0.836	10.584
a. Dependent Variable: RSESTotal; F = 3.529; R square = 0.187; Adjusted R square = 0.134; model *p*-value = 0.022								
**Biology–Clinical Biochemistry/Genetics Modules combined** (variables were coded as gender: male = 1, and female = 2; semester: 1st semester = 1, and >1st semester = 2; secondary education: general high school = 1, and vocational high school = 2; subject: biology–clinical biochemistry = 1, and genetics = 2).								
1	(Constant)	29.572	3.925		7.535	<0.001	21.835	37.309
	Gender	3.022	1.374	0.147	2.200	**0.029**	0.314	5.730
	Subject	−3.758	1.290	−0.197	−2.914	**0.004**	−6.300	−1.216
a. Dependent Variable: PSSTotal; F = 3.728; R square = 0.067; Adjusted R square = 0.049; model *p*-value = 0.006								
1	(Constant)	3.808	0.287		13.273	<0.001	3.242	4.373
	Subject	0.188	0.094	0.138	1.997	**0.047**	0.002	0.374
a. Dependent Variable: AGQ_MA; F = 1.089; R square = 0.020; Adjusted R square = 0.002; model *p*-value = 0.363								
1	(Constant)	3.121	0.394		7.920	<0.001	2.344	3.897
	Gender	0.458	0.138	0.223	3.319	**0.001**	0.186	0.730
a. Dependent Variable: AGQ_MAV; F = 3.563; R square = 0.064; Adjusted R square = 0.046; model *p*-value = 0.008								
1	(Constant)	2.583	0.454		5.684	<0.001	1.687	3.479
	Gender	0.387	0.159	0.165	2.430	**0.016**	0.073	0.700
a. Dependent Variable: AGQ_PAV; F = 1.974; R square = 0.036; Adjusted R square = 0.018; model *p*-value = 0.100								
1	(Constant)	22.854	2.703		8.454	<0.001	17.525	28.183
	Secondary_Education	2.548	0.930	0.191	2.738	**0.007**	0.714	4.382
a. Dependent Variable: RSESTotal; F = 2.352; R square = 0.043; Adjusted R square = 0.025; model *p*-value = 0.055								

Bold is to highlight statistically significant associations.

## Data Availability

The raw data supporting the conclusions of this article will be made available by the authors on request.

## Public Involvement Statement

There was no public involvement in any aspect of this research.

## Guidelines and Standards Statement

This manuscript was prepared in accordance with the STROBE (Strengthening the Reporting of Observational Studies in Epidemiology) guidelines for cross-sectional studies.

## Abbreviations

The following abbreviations are used in this manuscript:

AGQ-R	Achievement Goal Questionnaire-Revised
PSS-14	Perceived Stress Scale-14
AGQ MA	Mastery-approach goals
AGQ MAV	Mastery-avoidance goals
AGQ PA	Performance-approach goals
AGQ PAV	Performance-avoidance goals
RSES	Rosenberg Self-Esteem Scale
SEM	Standard error of the mean
SD	Standard deviation
CI	Confidence interval
